# State Aid for the Working Man's Child

**Published:** 1918-08-24

**Authors:** 


### STATE AID FOR THE WORKING MAN'S CHILD.

### (FROM A CORRESPONDENT.)

According to a paragraph in the *Daily Telegraph* of August 6, the Executive of the National Union of Scottish Miners at Glasgow, under the presidency of Mr. Robert Smillie, agreed to a resolution favouring the endowment of motherhood by providing a State allowance of five shillings a week for each child born in a working man's family. What a working man is, is not defined in the resolution, but it probably includes miners, or it is not likely the resolution would have been passed. Miners are about as highly paid as any manual labourers. Such of them as choose to work hard and steadily may earn £300 a year or over, and many, taking much leisure time for sport and amusement, make £200 to £250 a year.

It is a little hard to see why a class that earn so much should have the expenses of paternity taken off their shoulders by the taxing of the small shopkeepers, the clergy of all denominations, the professional men, the small owners of property, etc., unless it is proposed that all persons with less than a miner could earn if he chose to put his back into his work shall have a dole from other people's charity, voluntary or involuntary. It is a sign of bad omen when highly paid men are willing to cadge from the community for the keep of their children. Where is the sturdy independence of the horny-handed son of toil? Character is hereditary and parental teaching develops it. Are the people who are always holding out their hands for State aid likely to breed and bring up valuable citizens for the State?

It is possible that some day the State may pay women to breed children for it, and it might be right so to do; an honourable service for which a woman should be proud to receive pay. But it is hardly likely that any State is going for long to allow any man, miner or other, to gratify his lust with any woman he likes to select, and then save him the expense of providing for the result. If the State is to pay for the children, the State will demand the right to say what women are to bear the children, and what men may father them. Will such interference please the sturdy, independent miners? Or do they really expect any useless man and any useless woman to be allowed to breed all the children they like and the community to relieve them of the burden, and even, if the family comes fast enough, to pay the parents enough to keep them in idleness? Such resolutions sound very nice to the shallow thinkers who frame them and applaud them, but in what would such a policy end? State pay means State interference in the end.

